# Characterization of Mobile *Staphylococcus equorum* Plasmids Isolated from Fermented Seafood That Confer Lincomycin Resistance

**DOI:** 10.1371/journal.pone.0140190

**Published:** 2015-10-08

**Authors:** Jong-Hoon Lee, Do-Won Jeong

**Affiliations:** 1 Department of Food Science and Biotechnology, Kyonggi University, Suwon, 443–760, Republic of Korea; 2 Department of Food Science and Biotechnology, Shinansan University, Ansan, 425–792, Republic of Korea; Leibniz-Institute DSMZ, GERMANY

## Abstract

The complete nucleotide sequences of lincomycin-resistance gene (*lnuA*)-containing plasmids in *Staphylococcus equorum* strains isolated from the high-salt-fermented seafood jeotgal were determined. These plasmids, designated pSELNU1–3, are 2638-bp long, have two polymorphic sites, and encode typical elements found in plasmids that replicate via a rolling-circle mechanism including the replication protein gene (*rep*), a double-stranded origin of replication, a single-stranded origin of replication, and counter-transcribed RNA sequence, as well as *lnuA*. Plasmid sequences exhibit over 83% identity to other *Staphylococcus* plasmids that harbor *rep* and *lnuA* genes. Further, three pairs of identified direct repeats may be involved in inter-plasmid recombination. One plasmid, pSELNU1, was successfully transferred to other *Staphylococcus* species, *Enterococcus faecalis*, and *Tetragenococcus halophilus* in vitro. Antibiotic susceptibility of the transconjugants was host-dependent, and transconjugants maintained a lincomycin resistance phenotype in the absence of selective pressure over 60 generations.

## Introduction

The widespread use of antibiotics that prevent or treat bacterial infection has contributed to an increase of antibiotic-resistant bacteria in the environment, clinical settings, and in the food chain. Togay et al. [1] have reported that 32% and 24% of *Enterococcus faecium* isolates found in Turkish fermented foods exhibit resistance to tetracycline and kanamycin, respectively. Among *Lactobacillus plantarum* isolates found in Italian fermented dry sausages, approximately 50% exhibited phenotypic resistance to erythromycin [2], while almost half of coagulase-negative staphylococci (CoNS) isolates found in traditionally fermented sausages showed phenotypic resistance to at least one antibiotic [3].

Fermented foods act as reservoirs and vehicles for large populations of living bacteria and have been proposed as a possible source of antibiotic-resistant bacteria [4–6]. In support of this hypothesis, Gazzola et al. [7] demonstrated horizontal transfer of conjugative plasmids and antibiotic resistance genes among food microbiota within a sausage fermentation model system. To address these concerns, a report published by the US Centers for Disease Control and Prevention [8] to address the spread of food-borne pathogens with acquired antibiotic resistance genes has warned that the threat of antibiotic resistance, including the spread of antibiotic-resistant bacteria from foods to humans, is imminent.

We recently used cultivable bacterial community analysis to identify *Staphylococcus equorum* as the predominant bacterial species in jeotgal, a Korean high-salt-fermented seafood [9]. The safety and technological properties of 126 jeotgal *S*. *equorum* isolates were further assessed to select for safe and efficient starter cultures [10]. Among the 126 isolates, PCR analysis identified four strains that possessed the lincomycin resistance gene (lincosamide *O*-nucleotidyltransferase gene, *lnuA*). All amplified *lnuA* gene fragments showed nearly identical sequence identity, even though the harboring strains were isolated from two different types of jeotgal. Furthermore, all *lnuA*-harboring strains exhibited resistance to other antibiotics as well, with varying phenotypic resistance patterns. These results suggest that these *S*. *equorum* isolates acquired the *lnuA* gene from the environment prior to their proliferation in jeotgal.

In this study, we characterized the *lnuA*-containing plasmids purified from these strains and assessed their in vitro transferability to demonstrate the possibility of horizontal antibiotic resistance gene transfer within food-involved bacteria.

## Materials and Methods

### Bacterial strains and culture conditions

Four *S*. *equorum* isolates (KS1022, KS1030, KS3044, and KM1031) harboring the *lnuA* gene were used to characterize the *lnuA*-encoded plasmids (Table 1) and have been described previously [10]. Strain KS1030 was used to assess gene transferability. Recipient strains in the plasmid transfer experiment were selected from our stock cultures and were isolated from traditional Korean fermented foods (Table 1) [10, 11]. The *Staphylococcus aureus* USA300 LAC strain is a previously described clinical isolate [12, 13] that was adopted as a recipient strain for this study. All strains were primarily cultured in tryptic soy agar (TSA) and tryptic soy broth (TSB) at 30°C for 24 h to maintain their phenotypic traits.

**Table 1:** Bacterial strains examined in this study and corresponding MICs.

Strain	Origin	Phenotype	MIC (mg/l) against antibiotics					Reference
			Lin	Clin	Tet	Amp	Van	
*S*. *equorum* KS1022	Saeu-jeotgal	Ery^R^, Lin^R^, Pen^R^	512	16				[10]
*S*. *equorum* KS1030	Saeu-jeotgal	Lin^R^, Pen^R^	512	32				[10]
*S*. *equorum* KS3044	Saeu-jeotgal	Lin^R^, Liz^R^, Pen^R^	512	32				[10]
*S*. *equorum* KM1031	Myeolchi-jeotgal	Ery^R^, Chl^R^, Lin^R^, Pen^R^	1024	128				[10]
*S*. *equorum* C3056	Saeu-jeotgal	Tet^R^	0.5	0.5	32			[10]
*S*. *saprophyticus* KM1053	Myeolchi-jeotgal	Tet^R^, Pen^R^	0.5	0.5	32			[44]
*S*. *aureus* USA300 LAC	Human	Amp^R^	0.5	0.5		>100		[12, 13]
*E*. *faecalis* 7AME16	Meju	Van^R^	0.5	0.5			512	[11]
*T*. *halophilus* 7BDE12	Doenjang	Tet^R^	0.5	0.5	64			[11]

Abbreviations: Ery, erythromycin; Chl, chloramphenicol; Clin, Clindamycin; Lin, lincomycin; Liz, linezolid; Pen, penicillin G; Tet, tetracycline; Amp, ampicillin; Van, vancomycin.

### Identification and sequence analysis of *S*. *equorum* plasmids

Plasmid DNA was extracted with an Inclone™ plasmid mini prep kit (InClone, Daejeon, Korea) after lysostaphin (40 μg/ml) treatment at 37°C for 30 min to lyse cell walls. Plasmid DNA was concentrated using a Centricon centrifugal filter device YM-50 (Millipore, Bedford, USA). Concentrated samples were separated on 1% (w/v) agarose gels. The smallest bands were extracted using a QIAquick Gel Extraction kit (Qiagen, Hilden, Germany) and were identified by restriction enzyme analysis with *Spe*I and *Hin*dIII. The restricted fragments were cloned into corresponding pUC18 sites and the sequences were determined. Full plasmid sequences were confirmed and determined by PCR. PCR amplification was performed with primers designed to correspond to determined sequences on each plasmid using a T3000 Thermocycler (Biometra, Gottingen, Germany). Each PCR reaction mixture contained 10 ng template DNA, 0.5 μM each primer, and 1 U *Taq* polymerase (InClone), according to the manufacturer’s recommendations. Samples were preheated at 95°C for 5 min, amplified with 30 cycles by denaturing at 95°C for 1 min, annealing at 58°C for 1 min, and extending at 72°C for 1 min. PCR products were purified using a PCR purification system kit (SolGent, Daejeon, Korea) and DNA sequences were determined by a custom service provided by GenoTech (Daejeon, Korea). DNA and amino acid sequence data analyses were performed using the Lasergene sequence analysis software package (Dnastar, Madison, USA). Sequence similarities were identified using the BLASTX program at the National Center for Biotechnology Information website (http://blast.ncbi.nlm.nih.gov/).

### Plasmid transfer experiment

To determine the transferability of the *lnuA*-harboring plasmid, the *S*. *equorum* KS1030 strain was mated with different recipient strains using the broth mating method [14]. Recipient strains were lincomycin-sensitive and conferred specific antibiotic resistance to facilitate transconjugant selection. Logarithmic phase donor cells cultured in Mueller-Hinton (MH) broth (BD Diagnostic Systems, Sparks, USA) were mixed with logarithmic phase recipient cells cultured in MH broth at a 1:10 ratio and incubated at 30°C for 3 h. The mixture was spread onto the surface of TSA plates supplemented with 30 mg/l lincomycin and other appropriate antibiotics. Other antibiotics were used at the following concentrations: ampicillin, 50 mg/l; tetracycline, 10 mg/l; and vancomycin, 10 mg/l. Transconjugants were selected after incubation at 30°C for 24 h, and were confirmed by colony PCR with primers corresponding to the *lnuA* gene [10, 15]. Recipient traits of transconjugants were confirmed by 16S rRNA gene sequence analysis.

### Minimum inhibitory concentration (MIC) determination

Antibiotic MICs were determined by the broth microdilution method as described previously [16]. Each antibiotic was prepared with serial twofold working dilutions in deionized water and the final concentration of each antibiotic in one 96-microwell plate ranged between 0.5 and 4096 mg/l. Bacterial strains were cultured twice in TSB and matched a McFarland 0.5 turbidity standard (bioMérieux, March L'Etoile, France). Each suspension was diluted a further 1:100 in cation-adjusted MH broth to achieve an adequate inoculum concentration. The final inoculum density was 5 × 10^5^ colony-forming units (cfu)/ml per well on 96-microwell plates. Microwell plates were incubated at 35°C for 18 h and incubated 24 h to determine the MIC for vancomycin. The MIC of each antibiotic was recorded as the lowest concentration where no growth was observed in the wells after incubation for 18h or 24 h. MIC results were confirmed by at least three independently performed tests.

### Evaluation of plasmid stability

The segregational stability of the *lnuA*-encoded plasmid in *S*. *equorum* KS1030 and transconjugants was determined as described previously by Roberts et al. [17]. Briefly, a single colony was inoculated into selection-pressure-free TSB and cultured at 30°C for 24 h. The saturated culture was diluted to 10^−3^ in fresh TSB, and this same dilution was repeated into fresh TSB every 24 h. Each culture sample was plated on TSA through serial dilution and incubated overnight at 30°C. Individual colonies were picked and streaked onto lincomycin (30 mg/l) TSA plates to check for lincomycin resistance.

## Results

### Nucleotide sequences of plasmids carrying *lnuA*

**Fig 1:**
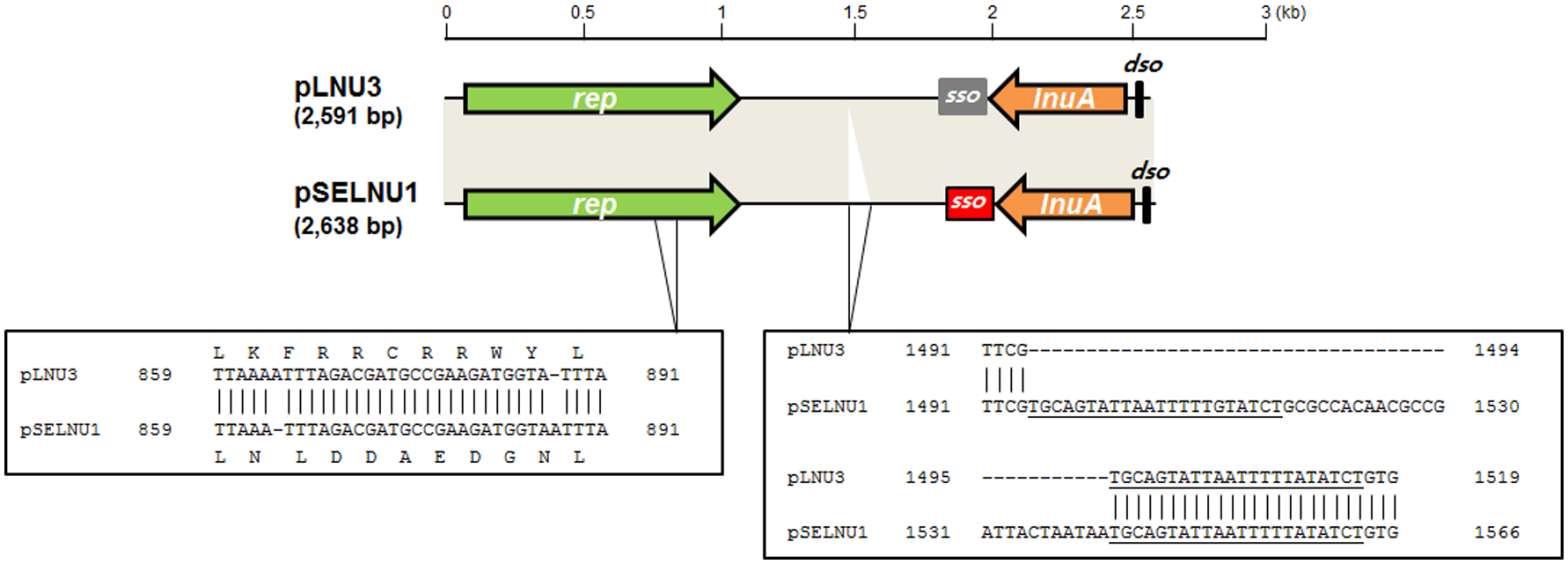
Gene structures of pLNU3 and pSELNU1. Shaded boxes correspond to sequences that exhibit 99% nucleotide sequence identity between pLNU3 and pSELNU1. Major sequence differences between both plasmids are identified as empty boxes. Amino acid changes in the Rep protein between both plasmids are also shown.

The *lnuA* gene sequences of four lincomycin-resistant strains isolated from fermented seafood were characterized in this study (Table 1). Sequences of partially amplified *lnuA* genes from these four lincomycin-resistant *S*. *equorum* strains are identical to that of the previously characterized *lnuA* gene on pLNU3 of the *Staphylococcus haemolyticus* coa101 strain [18]. In addition, all four *S*. *equorum* strains possess more than one plasmid and their smallest bands are commonly detected (S1 Fig). Restriction enzyme and sequence analyses show that the smallest common plasmid size is 2638 bp. Sequence analyses reveal that these plasmids contain elements that are typical of plasmids that replicate via a rolling-circle mechanism: the entire replication protein gene (*rep*), a double-stranded origin of replication (*dso*), a single-stranded origin of replication (*sso*), and counter-transcribed RNA (ctRNA) sequence, together with the *lnuA* gene (Fig 1 and S2 Fig). Plasmids purified from KS1022 and KS1030 have the same nucleotide sequence, and are thus designated pSELNU1. Plasmids purified from KS3044 and KM1031 harbor one- and two-nucleotide differences with pSELNU1, respectively, and are named pSELNU2 (G87T) and pSELNU3 (G87T and A1117T). Overall, the pSELNU plasmids exhibit 99% nucleotide sequence identity with pLNU3. Three polymorphic sites observed amongst these plasmids at positions 68, 87, and 1117 do not appear to affect plasmid function, while differences at positions 954 and 976 resulted in Rep proteins that differ by nine amino acids (Fig 1). To our knowledge, these altered sequences have not been reported to be associated with any key Rep protein functions to date. In addition, all pSELNU plasmids are longer than pLNU3 by 47 bp; this corresponds to a fragment inserted in the non-coding region between the *rep* and *lnuA* genes (Fig 1). This 47-bp fragment contains a direct repeat sequence of 22 bp (DR1) identified at the flanked sequence (S2 Fig). The nucleotide sequences of pSELNU1, pSELNU2, and pSELNU3 have been deposited in the GenBank database under accession numbers KP178913, KP178914, and KP178915.

### Characterization of pSELNU1

**Fig 2:**
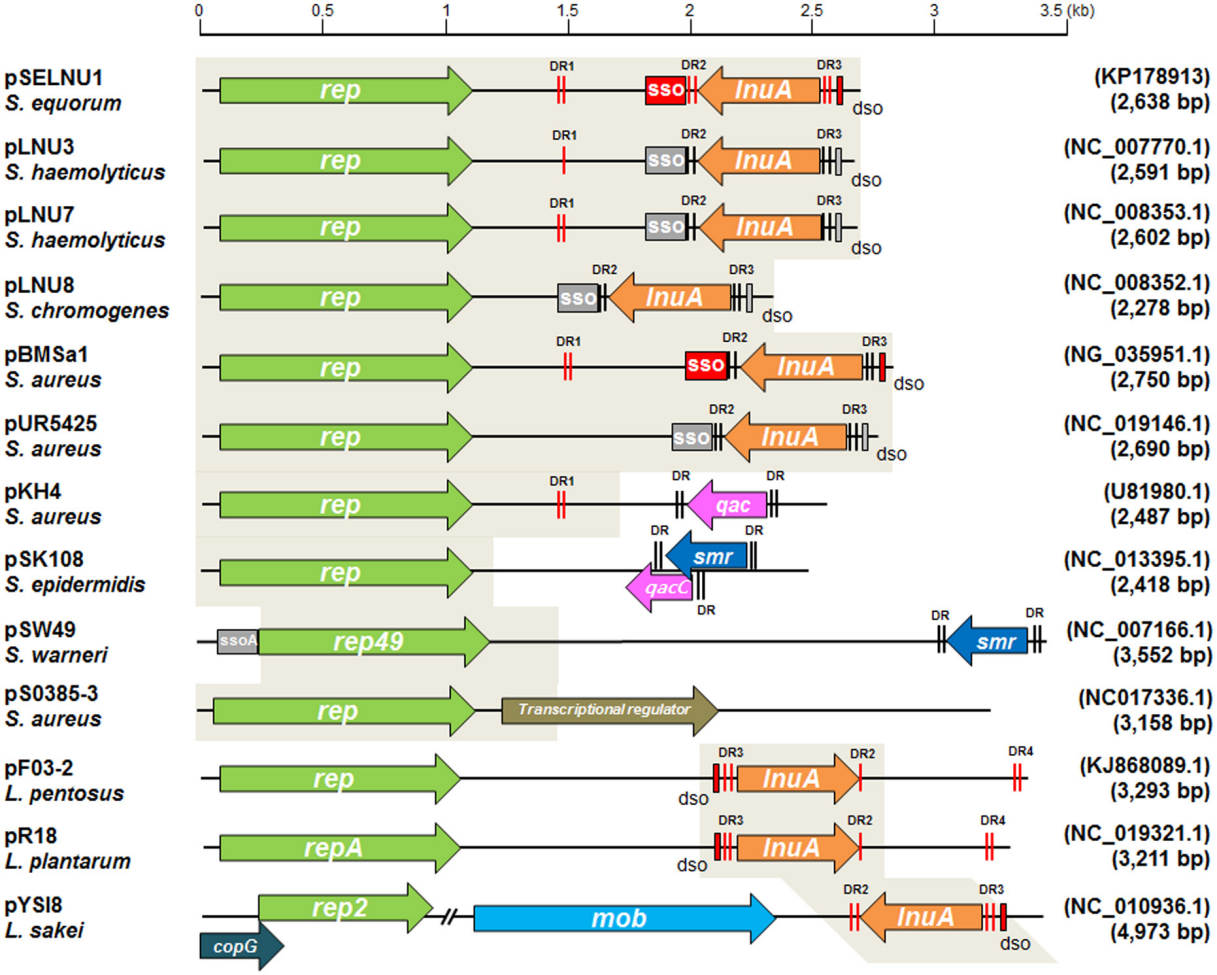
Small plasmid structures, *rep* and antibiotic/stress resistance genes. Shaded boxed regions possess over 80% nucleotide sequence identity with pSELNU1. *Dso* and *sso* regions are shown as color boxes and direct repeat sequences are indicated with vertical lines. All sequences identified in this study are indicated with red.

pSELNU1 contains two divergently transcribed open reading frames corresponding to *rep* and *lnuA* (Fig 1). The putative Rep protein encoded by pSELNU1 comprises 334 amino acids and exhibits 97% sequence identity with the Rep protein encoded on *S*. *haemolyticus* coa101 pLNU3 [18], 96% identity with the Rep protein encoded on *S*. *aureus* ST398 pS0385-3 [19], and 47% identity with the Rep2 protein encoded on a *Lactobacillus sakei* pYSI8 plasmid [20] (Fig 2). The putative LnuA protein encoded by pSELNU1 comprises 161 amino acids and it exhibits 100% and 96% sequence identities with *S*. *haemolyticus* coa101 pLNU3 [18] and *S*. *aureus* ST398 pBMSa1 [21], respectively. Furthermore, the LnuA protein on pSELNU1 exhibits 94%–98% homology with plasmids pF03-2, pR18, and pYSI8 from *Lactobacillus* species, while the pSELNU1 Rep protein exhibit 47%–60% homologies with the same plasmids (Fig 2).

Rep proteins involved in rolling-circle replication have been detected in various Gram-negative and Gram-positive bacterial plasmids and they have several conserved protein motifs [22, 23]. We detected three conserved motifs (G, T, and HUH) in the pSELNU1 Rep protein (S2 Fig). Rep protein has been known to recognize the *dso*-containing nick site [24] and the pSELNU1 *dso* site is located upstream of the *rep* gene start codon (positions: 2553–2570). Also, pSELNU1 possess a recombination site B sequence (RSB) at nucleotides 1850–1866 (5′-TTATGCCGAGAAAATTT-3′) and a 6-bp consensus sequence (CS-6) at nucleotides 1917–1922 (5′-TAGCGT-3′), which are the typical conserved sequences of *sso* [25]. In addition, we detect two putative hairpin structures at positions 1852–1917, when a RNA secondary structure prediction program is used (http://rna.urmc.rochester.edu). The *sso* are known to have several inverted repeats that can generate stem-loop structures, which are known to be important in lagging strand initiation [26]. We observe a ctRNA sequence in pSELNU1 (35–80) that is located upstream of *rep* and that is transcribed in the opposite direction. It comprises a putative inverted repeat sequence that may regulate *rep* transcription by binding to a paired sequence [27]. Indeed, we detect a putative paired sequence within the *rep* gene at position 333–337. A putative promoter for ctRNA that consists of a -35 region (5′-TTGATT-3′, 129–124) and a -10 region (5′-TATGAT-3′, 106–101) is also present. Lastly, two pairs of direct repeats, DR2 (5′-AATAAAGCAAT-3′) and DR3 (5′-AAACGAGTT-3′), are located at the junctions between *sso* or *dso* and the *lnuA* gene in pSELNU1.

### Horizontal transfer of *lnuA* by conjugation

Intraspecific transfer of pSELNU1 was investigated by mating strains *S*. *equorum* KS1030 and *S*. *equorum* C3056, and by selecting for tetracycline resistance conferred by the *tetK* gene. Transconjugants showing phenotypic lincomycin and tetracycline resistance were detected at a frequency of 5.4 × 10^−5^ (Table 2). When interspecific transferability was tested by mating *Staphylococcus saprophyticus* KM1053 and *S*. *aureus* USA300 LAC, the plasmid was only transferred to *S*. *saprophyticus* at a frequency of 7.7 × 10^−6^. It was also successfully transferred to *Enterococcus faecalis* 7AME16 and *Tetragenococcus halophilus* 7BDE12, at frequencies of 1.3 × 10^−6^ and 4.6 × 10^−6^, respectively (Table 2).

**Table 2:** In vitro transfer of *lnuA* from *S*. *equorum* to Gram-positive recipient strains.

Mating organism		Cell count (cfu/ml)			Transfer rate (T/R)	MIC of transconjugant			
Donor strain	Recipient strain	Donor	Recipient	Transconjugant^a^		Lin	Clin	Tet	Van
*S*. *equorum* KS1030	*S*. *equorum* C3056	3.3 × 10^8^	1.7 × 10^7^	9.3 × 10^2^	5.4 × 10^−5^	>512	>32	>16	
*S*. *equorum* KS1030	*S*. *saprophyticus* KM1053	2.1 × 10^8^	4.6 × 10^7^	3.3 × 10^2^	7.7 × 10^−6^	>512	>32	>32	
*S*. *equorum* KS1030	*S*. *aureus* USA300 LAC	1.6 × 10^8^	1.2 × 10^7^	-					
*S*. *equorum* KS1030	*E*. *faecalis* 7AME16	4.5 × 10^8^	1.1 × 10^7^	2.0 × 10^1^	1.8 × 10^−6^	>256	>1024		>512
*S*. *equorum* KS1030	*T*. *halophilus* 7BDE12	2.2 × 10^8^	2.1 × 10^7^	1.3 × 10^2^	6.9 × 10^−6^	>256	>1024	>64	

Cell counts were repeated three times independently and the mean values of the replicates are presented. R, recipient; T, transconjugant.

^a^ Transconjugants were confirmed by the phenotypic resistance and 16S rRNA gene sequence analysis.

### Phenotypic lincomycin resistance of transconjugants

MICs for lincomycin and clindamycin of the donor strain *S*. *equorum* KS1030 harboring pSELNU1 are 512 and 32 mg/l, respectively (Table 1). The *S*. *equorum* C3056 and *S*. *saprophyticus* KM1053 transconjugants exhibit similar MICs (Table 2). However, when pSELNU1 is transferred to *E*. *faecalis* 7AME16 and *T*. *halophilus* 7BDE12, transconjugants exhibit lower lincomycin resistance and higher clindamycin resistance than the donor strain, while vancomycin and tetracycline resistance of recipient strains remains unchanged after acquisition of pSELNU1.

### Segregational and structural stability of pSELNU1

The segregational and structural stability of pSELNU1 in donor and transconjugant strains was examined to validate the stability of the pSELNU1 replication system in various hosts. The plasmid remained in donor and recipient strains after 60 generations in the absence of lincomycin, with the exception of *T*. *halophilus*, in which approximately 10% of transconjugants lost the pSELNU1 plasmid after 60 generations. Importantly, the plasmid profile of the donor strain after 60 generations is the same as the original profile, without any obvious alterations in size for any plasmids examined (data not shown).

## Discussion

Fermentation improves the nutritional, organoleptic, and functional properties of raw food materials via microbial activity. In general, traditionally fermented foods are produced in household units and spontaneous fermentation can often result in undesirable final products. Inoculation with a small quantity of back-slop can accelerate the initial phase of fermentation and can result in the promotion of desirable changes during the whole fermentation process. The application of select starter cultures remains another viable option to obtain quality fermentation products. However, starter cultures can act as reservoirs for antibiotic resistance that can be transferred to possibly pathogenic bacterial species, thereby complicating the treatment of disease or infection and leading to the spread of antibiotic-resistant bacteria [28]. Thus, starter cultures should be verified for antibiotic resistance gene transferability to prevent the spread of virulent determinants in food ecosystems.

The spread of antibiotic resistance among bacteria depends on horizontal gene transfer mechanisms that use mobile elements such as plasmids and transposons [29]. However, studies examining antibiotic resistance genes and transfer mechanisms for food-involved bacteria are rarely reported when compared with studies involving medically important pathogens. If food-involved bacteria are considered, primary objectives tend to focus on the identification of erythromycin and tetracycline resistance genes in lactic acid bacteria, largely to prevent infection in dairy farm environments [30–32]. Although lincomycin has been used to prevent infection in dairy cows and lincomycin resistance has been reported in high frequencies (36.4%) in CoNS from fermented foods [33], very few studies have examined lincomycin resistance genes in fermented foods [34].

Interestingly, the *lnuA*-containing pSELNU plasmids exhibit nearly identical DNA sequence and structural homology with previously characterized pLNU3, regardless of their geographical isolation point. The pSELNU plasmids are also homologous to several other characterized *Staphylococcus* plasmids that encode small and simple structures, the *rep* gene and resistance genes (Fig 2) [35]. More specifically, previously identified *Staphylococcus* plasmids encode genes such as the quaternary ammonium compounds resistance protein (*qac*), multidrug resistance efflux protein (*smr*), or putative transcriptional regulators [19, 36, 37]. Other plasmids with small and simple structures (pF03-2, pR18, and pYSI8) encoding *rep* and *lnuA* have been identified in *Lactobacillus* species as well, while exhibiting 47%–60% identity at the *rep* locus and 94%–98% identity at the *lnuA* locus in *Staphylococcus* species. Therefore, we hypothesize that small *rep*-containing plasmids present in staphylococci or lactobacilli may acquire antibiotic or stress resistance genes by recombining under specific environmental pressures.

We also identified three pairs of direct repeats in pSELNU plasmids. These elements have been identified not only in *lnuA*-carrying plasmids, but in resistance gene-containing plasmids purified from staphylococci and lactobacilli as well. It is possible that the cassette-like structures of these repeats contribute to the exchange of different resistance genes between plasmids and inter-plasmid recombination [38]. This assumption has been extensively supported by the identification of identical resistance genes that occur in combination with different replication genes or vice versa [39–41]. The conserved direct repeat sequences DR2 and DR3, are schematically represented in Fig 2. Stochastically, the cassette-like structure of these repeats can position *lnuA* in two directions, although most *lnuA* genes in plasmids are divergently transcribed with *rep* (pF03-2 and pR18 are exceptions to this statement). It is possible that divergent transcription of *lnuA* and *rep* may allow for the distribution of genetic information to both DNA strands, while allowing for termination of *lnuA* transcription. If *lnuA* and *rep* were transcribed in the same direction, the *dso* located downstream of *lnuA* may be affected by termination of the *lnuA* transcript. Interestingly, two plasmids pF03-2 and pR18, both of which harbor *rep* and *lnuA* in same direction, possess a conserved DR2 and an additional direct repeat pair DR4 downstream of *lnuA*. DR2 might not recombine in this context, but the direct repeat pair DR4 could recombine with a *rep* plasmid in lactobacilli species. Recombination mediated by DR3 and DR4 could allow for the transcription of *lnuA* regardless of direction because sufficient terminal sequences are secured without inhibiting the neighboring sequence function. Finally, we note that the direct repeat pair DR1 identified in pSELNU1 is also found in several *Staphylococcus* plasmids. Although DR1 is longer than DR2, DR3, and DR4, it is entirely possible that its function is similar to that of the other direct repeat sequences.

Lincomycin has been used to prevent bacterial infection and treat bovine subclinical mastitis caused by CoNS species. Lincomycin has also been used to prevent bacterial infections in aquaculture, potentiating the development of lincomycin-resistant bacteria in a marine environment. *Staphylococcus* species have been isolated from sea water [42] and we isolated *S*. *equorum* strains harboring *lnuA* from two types of jeotgal made from anchovies and tiny shrimps [10]. The intraspecific and interspecific transferability of pSELNU1 suggests that this plasmid can be spread in marine environments by encountering *Staphylococcus* species in the same niche. As such, we demonstrated that horizontal transfer of pSELNU1 from *S*. *equorum* to tetracycline-resistant *S*. *equorum*, *S*. *saprophyticus*, and *T*. *halophilus*, and vancomycin-resistant *E*. *faecalis* is possible in a laboratory setting. However, horizontal transfer to a *S*. *aureus* recipient strain did not occur when the recipient was a clinical isolate. Clinical *S*. *aureus* isolates are reputedly difficult to manipulate genetically and horizontal gene transfer is blocked by their restriction-modification system [43]. This limited gene transfer to pathogenic *S*. *aureus* may ease concerns over the spread of antibiotic resistance from food fermentation starter cultures to pathogenic bacteria. However, high sequence identities of *rep* genes (93%) and their deduced amino acids (96%) in pSELNU1 and pS0385-3 (accession No. AM990995.1) suggest that replication of pSELNU1 in *S*. *aureus* is theoretically possible. Moreover, successful transfer of pSELNU1 to *E*. *faecalis* and *T*. *halophilus* indirectly suggests that the existence of other unknown plasmids harboring similarly encoded structures is entirely plausible, and that pSELNU1 may spread to Gram-positive bacteria. Contrarily, the plasmid will likely not be transferred to Gram-negative bacteria, as they are generally not considered part of the lincomycin treatment spectrum.

As of yet, the exact mechanism underlying pSELNU1 transfer remains unknown and it cannot be explained by plasmid sequence analysis, as conjugative transfer elements have not yet been found. During conjugative gene transfer, conjugative elements such as the relaxase gene facilitate transfer of genetic material upon binding to single-stranded DNA. With this, pSELNU1 may be transferred with the assistance of unknown determinants encoded on other plasmids or chromosomal DNA.

The use of starter cultures that harbor pSELNU plasmids can potentially spread lincomycin resistance to other Gram-positive bacteria in the food microbial community, resulting in a human health hazard. Thus, a better understanding of the molecular basis underlying this gene transfer mechanism is required in successive research to prevent the spread of pSELNU plasmids.

## Supporting Information

S1 Fig Plasmid profiles of *S*. *equorum* isolates harboring the lincomycin resistance gene. Plasmids were extracted from the following strains: 1, KS1022; 2, KS1030; 3, KS3044; 4, KM1031. The ladder indicates the positions of DNA size markers.
(TIF)

S2 Fig Nucleotide sequence of pSELNU1. All elements involved in the double-stranded origin of replication (*dso*) are blue. The single-stranded origin of replication (*sso*) region containing a recombination site B sequence (RSB) and a 6-bp consensus sequence (CS-6) are shown as black boxes. Putative promoter regions of the *rep* and ctRNA genes are highlighted in red. The putative ctRNA stem-loop structure and inverted repeat sequences are indicated as differently colored horizontal arrows. The corresponding paired sequences are indicated with asterisks. The conserved amino acids of the Rep protein, the G, T, and HUH motifs, are shown in bold red letters. Direct repeats are highlighted in green.
(TIF)
