# Supplementary figures and images for: Characterization of Mobile Staphylococcus equorum Plasmids Isolated from Fermented Seafood That Confer Lincomycin Resistance
Source: PLoS One. 2015 Oct 8;10(10):e0140190. doi: 10.1371/journal.pone.0140190 (PMC4598088; doi:10.1371/journal.pone.0140190)

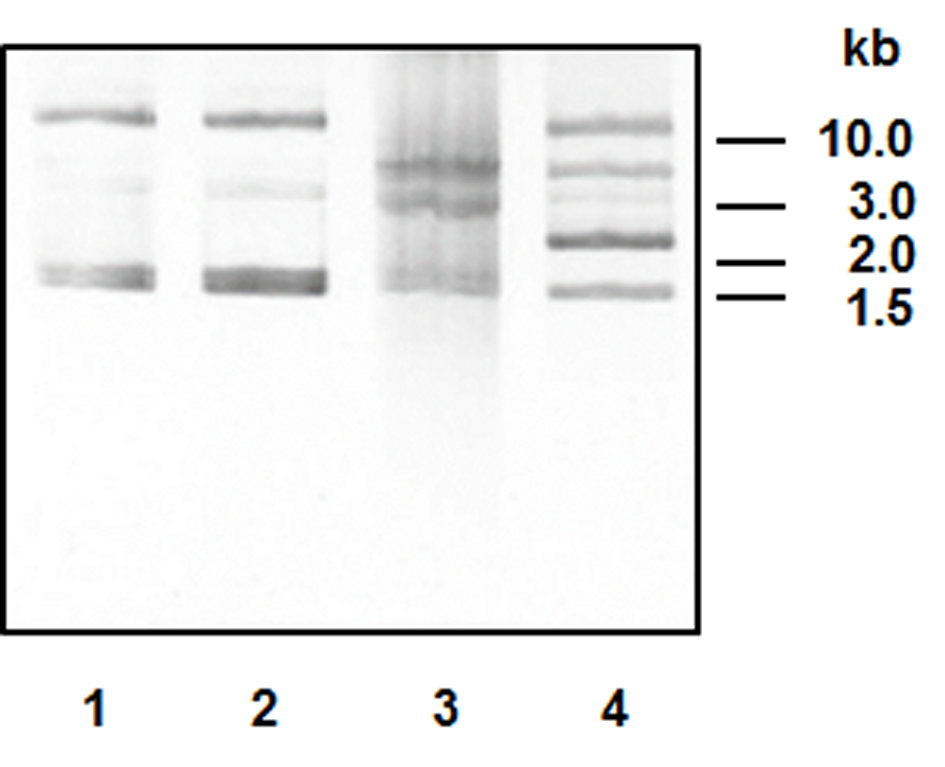

Supplement: S1 Fig — Plasmids were extracted from the following strains: 1, KS1022; 2, KS1030; 3, KS3044; 4, KM1031. The ladder indicates the positions of DNA size markers. (TIF) [file pone.0140190.s001.tif]

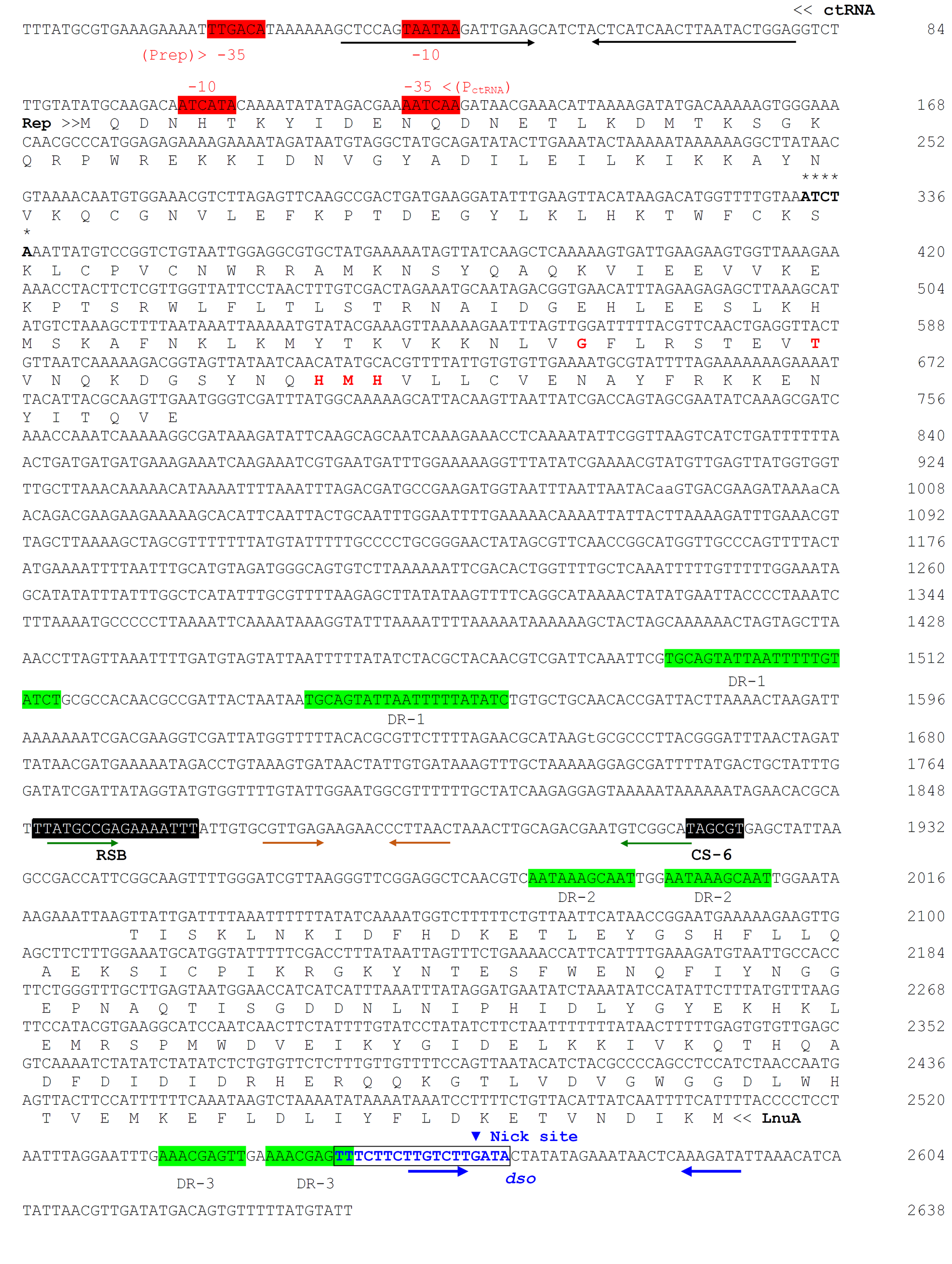

Supplement: S2 Fig — All elements involved in the double-stranded origin of replication (dso) are blue. The single-stranded origin of replication (sso) region containing a recombination site B sequence (RSB) and a 6-bp consensus sequence (CS-6) are shown as black boxes. Putative promoter regions of the rep and ctRNA genes are highlighted in red. The putative ctRNA stem-loop structure and inverted repeat sequences are indicated as differently colored horizontal arrows. The corresponding paired sequences are indicated with asterisks. The conserved amino acids of the Rep protein, the G, T, and HUH motifs, are shown in bold red letters. Direct repeats are highlighted in green. (TIF) [file pone.0140190.s002.tif]
